# Investigation on the Durability of E-Glass/Epoxy Composite Exposed to Seawater at Elevated Temperature

**DOI:** 10.3390/polym13132182

**Published:** 2021-06-30

**Authors:** Amir Hussain Idrisi, Abdel-Hamid I. Mourad, Beckry M. Abdel-Magid, B. Shivamurty

**Affiliations:** 1 Department of Mechanical Engineering, United Arab Emirates University, Al Ain 15551, United Arab Emirates; amir.hussain@uaeu.ac.ae; 2 National Water and Energy Center, United Arab Emirates University, Al Ain 15551, United Arab Emirates; 3 Mechanical Design Department, Faculty of Engineering, Helwan University, Cairo 11795, Egypt; 4 Department of Composite Materials Engineering, Winona State University, Winona, MN 5598, USA; bamagid@winona.edu; 5 Department of Mechanical and Manufacturing Engineering, Manipal Institute of Technology, Manipal 572104, India; shiva.b@manipal.edu

**Keywords:** glass fiber composite, elevated temperature, mechanical properties, microstructural analysis, durability, modeling

## Abstract

In this manuscript, the durability of the E-glass/epoxy composite was determined under a seawater environment. The effect of harsh environment was investigated in terms of seawater absorption, microstructure and degradation in mechanical properties. E-glass epoxy composite specimens were conditioned in gulf seawater at 23 °C, 65 °C and 90 °C for the period of 12 months. It was observed that the mass of the samples increased after the immersion of 12 months at 23 °C and 65 °C whereas it reduced at 90 °C. The salt deposition was observed at the surface of specimens without any crack for the seawater conditioning at 23 °C and 65 °C. The swelling and crack formation were significantly visible on the surface of the specimen immersed for 12 months at 90 °C. It indicates that the degradation mechanism accelerated at elevated temperature results fiber/matrix debonding. The tensile test indicates slight variation in the elastic modulus and reduction in strength of E-glass epoxy composite by 1% and 9% for specimens immersed at 23 °C and 65 °C respectively. However, at 90 °C, the tensile strength sharply decreased to 7% and elastic modulus significantly increased in the exposure of 12 months. A prediction approach based on a time-shift factor (TSF) was used. This model predicted that the strength retention of E-glass/Epoxy composite will be reduced to 7% in 450 years after immersion in seawater at 23 °C. Lastly, the activation energy for the degradation of the composite was calculated.

## 1. Introduction

In the past few years, several researchers have investigated the characteristics of composites in the marine environment. Glass Fiber-Reinforced Polymer (GFRP) composites are commonly used in automobile, aerospace and marine industries because of their high strength to weight ratio, stiffness and durability. Furthermore, glass fibers are simple to manufacture, economical, less fragile and high chemical resistant compared to carbon fiber. However, GFRPs face challenges in various environmental surroundings such as UV, seawater, alkaline and various other corrosive environmental conditions [1,2,3,4,5,6,7,8]. Polymer matrix composites trap water in voids at high temperatures and are often fused into hydroxyl radicals of the epoxy polymers [9,10,11,12]. The immersion in water results in deterioration in the physical properties of composites [13,14,15,16,17,18] which makes epoxy polymers inflate, breaks their bonds due to hydrolysis and further plasticizes [19,20,21]. The hot water environment contributes to small cracks within the polymer matrix due to water absorption and osmotic edge rupture [22,23,24], which increases the degradation of composites with immersion time. The durability of the structures also affected by the presence of defects on the surface of the polymer matrix composite before immersion. It can be regulated by determining the effect of the critical size of the osmotic blister [25]. Furthermore, the strength of the structure can be improved by repairing the structures with glass fiber-reinforced polymer [26,27]. For material effectiveness, it is essential to ensure the durability of FRP composites in the seawater environment. Nanofillers are one effective solution for closing any gaps and increasing the compositional intensity [28,29,30,31,32,33,34,35,36,37,38,39,40]. Several studies have been performed in different aging conditions for the performance of epoxy-based composites. It was previously observed that the flexural properties of FRP samples decreased due to seawater immersion [41,42]. Pavan et al. [43] investigated that tensile strength of glass/epoxy reduced from 200.83 MPa to 146.42 MPa, and 185.27 MPa for atmospheric and subzero temperatures aging for 3600 h. Yan et. al. [44] noticed a similar reduction in the mechanical properties of flax-fabric/epoxy composites. The tensile strength of the specimens reduced by 31.1%, 28.3% and 22.6% under the immersion of alkaline (5% NaOH) solution, seawater and water respectively. Silva et al. [45] studied the durability of GFRP laminates with epoxy resin by exposing the composites to saltwater at 30 °C, 50 °C and 65 °C for 5000 h. For the first 1500 h, the modulus behavior indicated that the prevailing damage mechanism was swelling. Then, between 1500 h and 2500 h, the elastic modulus behavior represented that plasticization was the predominant damage mechanism, especially for samples aged at 30 °C. Chakraverty et al. [46] studied the effect of seawater conditioning for up to 12 months (m) on the mechanical properties, such as interlaminar shear strength (ILSS), tensile stress and failure strain, elastic modulus and glass transition temperature (Tg) of GFRP composites. The tensile stress was reduced to 79% after immersion for 6 months but a slow recovery was observed, and the tensile strength rebounded to 84% after immersion for 12 months. Strain at rupture decreased by 12% and 20% after 6 months and 12 months of seawater immersion respectively. These reductions in values of stress and strain at rupture are due to the plasticization and swelling in the composite material. Chen Y. et al. [47] utilized the Arrhenius equation-based prediction model for predicting the durability of GFRP-reinforced concrete structures. The GFRP bars were immersed in a concrete solution at temperatures of 20 °C, 40 °C, and 60 °C. The short-term data were used for the accelerated aging tests. Similarly, Ali et al. [48] investigated the durability performance of the BFRP bars. The specimens were conditioned at 60 °C after different immersion periods (1000 h, 3000 h, and 5000 h). The prediction results show that the shear strength of BFRP bars will reduce by 19.8% and 23.0% after 150 years of immersion in the alkaline solution at 10 °C and 30 °C respectively. Dejke [49] investigated the durability of GFRP bars in alkaline solutions immersed at 20 °C, 40 °C, 60 °C and 80 °C, for a duration of 568 days. Samples were tested for tensile strength after removal from the solution. The time shift factor (TSF) approach was used for the prediction of tensile strength retention. It was observed that samples immersed at 60 °C for 1.5 years have the same tensile strength retention as for 50 years at 7 °C.

Table 1 summarizes the research related to E-glass/epoxy composite immersion at different temperatures. It is clear from the above literature review and research relevant to this topic mentioned in Table 1 that the data for harsh environment conditions (i.e., conditioning at 90 °C) are very lacking. Additionally, the harsh environmental conditioning is limited to a small exposure time. To the authors’ best knowledge, the durability evaluation of the fiber-reinforced polymer composites at a high temperature for one year has not been discussed in the literature.

The main objective of this work is to conduct an experimental investigation to evaluate the durability of E-glass/epoxy composite in harsh environmental conditions. Specimens were conditioned at different exposure temperatures (23 °C, 65 °C and 90 °C) in Gulf seawater for a period of 12 months. After exposure to the harsh environment, tensile strength, tensile strain and Young’s modulus of the specimen were determined. Furthermore, water absorption and scanning electron microscopy (SEM) analysis was conducted to investigate the damage mechanism of the composite. In addition to determining the degradation of the composite experimentally, these data were used to predict the durability of composites using Arrhenius-based models and the time-shift factor (TSF) approach.

## 2. Experimentation

**Figure 1:**
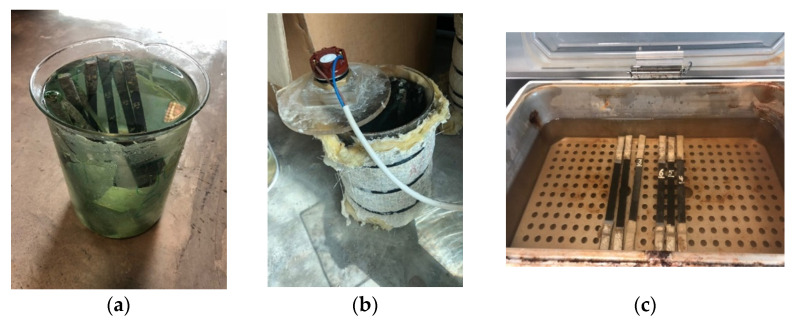
Conditioning of specimen at (**a**) 23 °C, (**b**) 65 °C and (**c**) 90 °C.

In this study, E-glass/epoxy composite reinforced with 52 vol% of glass fiber was utilized. The E-glass/epoxy was manufactured through a continuous lamination process and obtained from Gordon Composites, Inc. Samples with thickness of 3 mm for the tensile test were prepared as per ASTM Standard D-3039 [59]. Samples were conditioned at different exposure temperatures (23 °C, 65 °C and 90 °C) in Gulf seawater for the period of 12 months, as shown in Figure 1. Specimens conditioned at 23 °C and 65 °C were tested after every three months whereas the testing frequency was one month for the specimen immersed at 90 °C.

## 3. Results and Discussion

### 3.1. Water Absorption Test

The specimens were weighed prior to immersion in seawater at different temperatures. The specimens were taken out from the seawater tank and dried at room temperature for 24 h to determine the change in the mass. The percentage change in mass of specimen was calculated after drying as follows:

(1) $$M\;\left(\%\right)=\frac{M_{2}-M_{1}}{M_{1}}\times 100$$

where *M* (%) = mass change percentage, *M*_2_ = mass after drying (g), *M*_1_ = mass before immersion (g).

**Figure 2:**
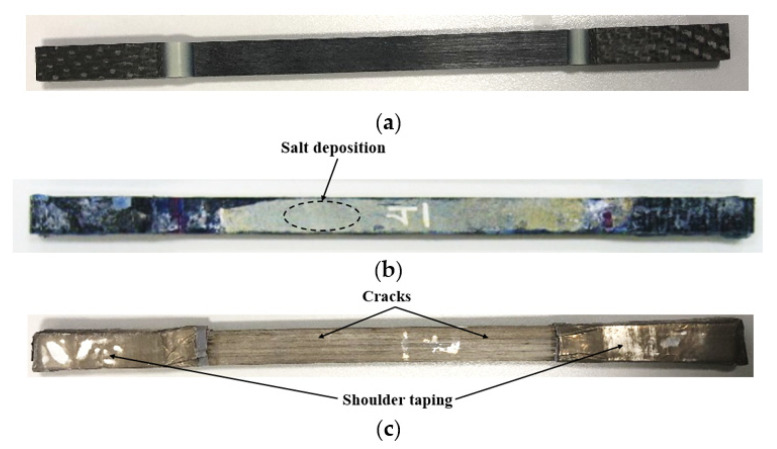
E-glass/epoxy specimen conditioned at various temperatures in seawater (**a**) Control sample, (**b**) 65 °C and (**c**) 90 °C.

Figure 2a shows the control specimen which is black in color with a clean surface. The effect of seawater and temperature was observed based on the crack on the surface of the specimen for different duration of time. In visual inspection, Figure 2b represents clear evidence of salt deposition on the surface of the samples immersed at 23 °C and 65 °C without any crack after 12 months of immersion in seawater. However, significant cracks were observed on the specimen immersed at 90 °C as shown in Figure 2c. The color of the specimen changed from black to gray for the specimen exposed at 90 °C whereas no change in color was observed for the specimen immersed at 23 °C and 65 °C. The change in the color of the specimen could be due to the micro-cracks which allow high-temperature seawater to penetrate through the layers of matrix and fiber results the removal of black pigment of the epoxy.

**Figure 3:**
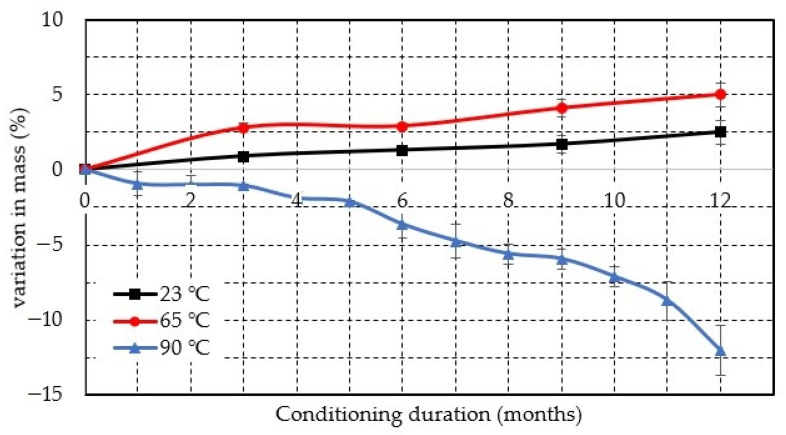
Effect of seawater conditioning on the mass of the specimen at various temperature.

Table 2 and Figure 3 show the percentage change in the mass of the specimen at different conditioning periods. The specimen mass increased with immersion time for the specimen conditioned at 23 °C and 65 °C but it reduced for the specimen immersed at 90 °C. The seawater absorption or increase in mass was 0.9%, 1.3%, 1.7% and 2.5% after 3 m, 6 m, 9 m and 12 m of conditioning at 23 °C respectively whereas 2.8%, 2.9%, 4.1% and 5% for seawater immersion at 65 °C. Similarly, Ray [60] observed an increase in water absorption with the increase in temperature from 60 °C to 70 °C. However, at 90 °C, the mass of the specimen dropped by 1%, 3.6%, 5.9% and 12% after immersion of 3 m, 6 m, 9 m and 12 m respectively. This may be due to the seawater that entered through the crack and degraded the fiber/matrix bonding. Furthermore, at this temperature, the epoxy and fiber glass may not fuse together properly and composite lost epoxy and seawater occupied the vacant place in the specimen. Wang et al. [61] noted a similar decrease in the mass of the composite at elevated temperatures.

### 3.2. Tensile Properties

The tensile test was performed at room temperature (300 K) using an MTS universal testing machine (MTS system corporation, Eden Prairie, MN, USA). The tests were conducted at a crosshead speed of 2 mm/min. Table 3 represents the tensile properties of the E-glass/epoxy composite immersed at 23 °C and 65 °C for 12 months. The tensile strength of control samples indicates the tensile properties of the samples tested without immersion. These results were used as a reference and comparison with conditioned samples to determine the effect of exposure temperature and duration. The tensile strength of the control specimen was 798 MPa. It was observed that the tensile strength of the specimen reduced by 1% from 798 MPa to 790 MPa and 9% from 798 MPa to 726 MPa after an immersion of 12 months in seawater at 23 °C and 65 °C respectively. The tensile properties of specimens conditioned in seawater at 90 °C for 12 months are shown in Table 4. The table indicates a sharp decrease in tensile strength by 92.7% from 798 to 57.3 MPa in 12 months of seawater conditioning at 90 °C.

**Figure 4:**
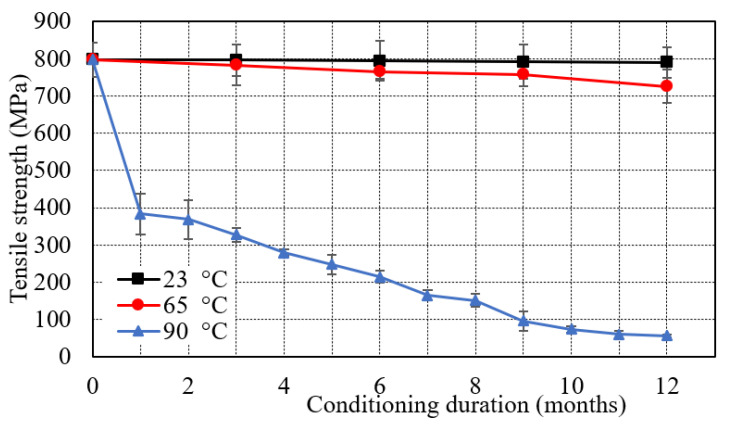
Effect of seawater immersion on tensile strength.

Figure 4 shows nearly a constant line for the specimen immersed at 23 °C. However, a small drop was observed in the tensile strength for the specimen immersed at 65 °C. At 90 °C, the tensile strength dramatically reduced to 384 MPa in 1 month which is 48.12% of the strength of control specimen. Furthermore, the tensile strength declined linearly from 384 to 57.3 MPa in the remaining period of 11 months which was almost 14.9%. 

**Figure 5:**
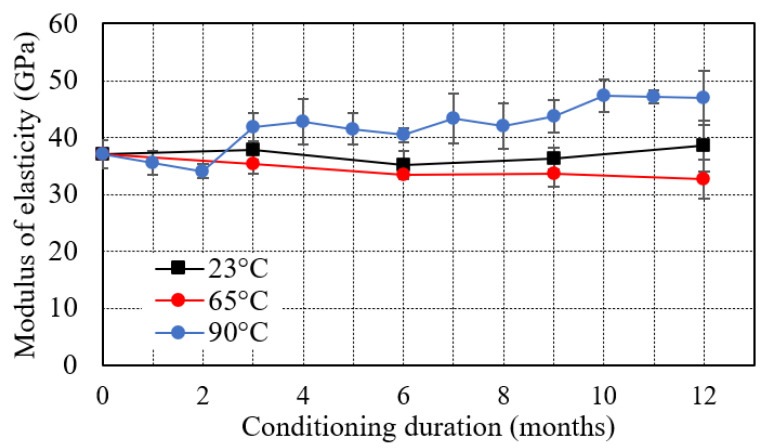
Effect of seawater immersion on modulus of elasticity.

Figure 5 indicates that seawater immersion slightly affected the elastic modulus of the samples conditioned at 23 °C and 65 °C. The elastic modulus varied from 37.1 GPa to 38.5 GPa and 32.7 GPa for specimen immersed for 12 months at 23 °C and 65 °C respectively. At an elevated temperature of 90 °C, elastic modus increased by 26.7% from 37.1 GPa to 46.9 GPa in the period of 12 months. The results indicate that the value of elastic modulus varied between 34.07 GPa and 47.38 GPa in the entire conditioning duration. The elastic modulus of the FRP specimen depends on the modulus of fibers. The immersion in seawater environment may not have any severe effect on the durability of the fibres, the modulus of GFRP has not suffered any obvious change after immersion [62].

**Figure 6:**
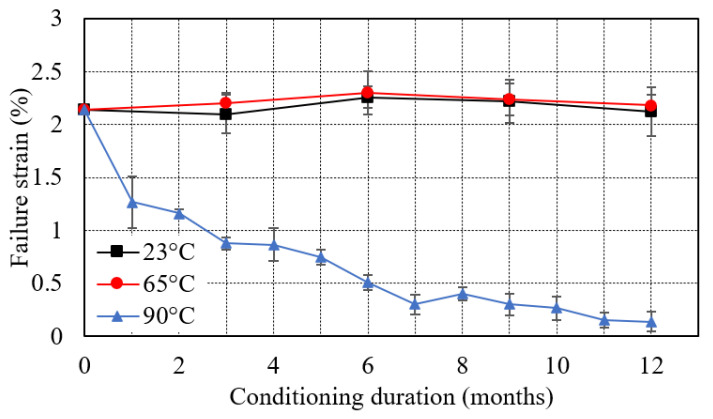
Effect of seawater immersion on failure strain (%).

The failure strain increased progressively from 2.14% to 2.26% after 6 months and then decreased to 2.12% after 12 months of immersion at 23 °C as shown in Figure 6. However, immersion at 65 °C results in a slight increase in failure strain from 2.14% to 2.3% after 6 months, then reduced to 2.18% after 12 months. For immersion at 90 °C, the opposite pattern was observed for failure strain. The failure strain dropped sharply from 2.14 to 1.27% in 1 month which is 59.2% of the control specimen. Thereafter, it declined gradually to 0.14% in the remaining 11 months with a comparatively slower rate. 

These data demonstrate that water absorption contributes to the epoxy matrix plasticization which affects the failure strain and tensile modulus. Over a duration of 12 months of use, the matrix becomes stiff and fragile, allowing failure strain to decrease and tensile modulus to increase dramatically. The tensile properties of the samples immersed at 90 °C significantly affected in 12 months of immersion. The failure strain of the samples reduced from 2.14% to 0.14% and tensile modulus increased from 37.1 GPa to 46.9 GPa. Merah et al. [54] observed a decrease in brittle fracture and failure strain due to prolonged immersion in seawater. 

**Figure 7:**
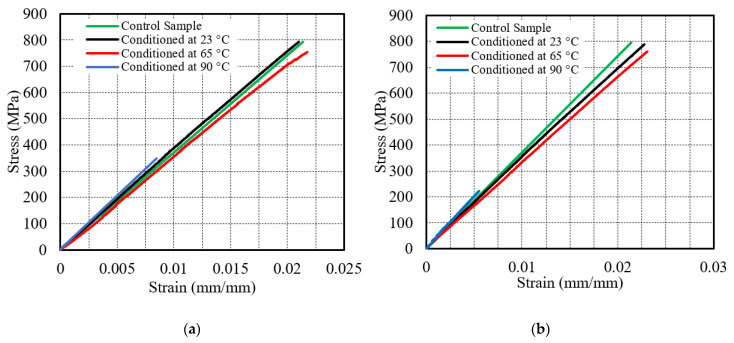

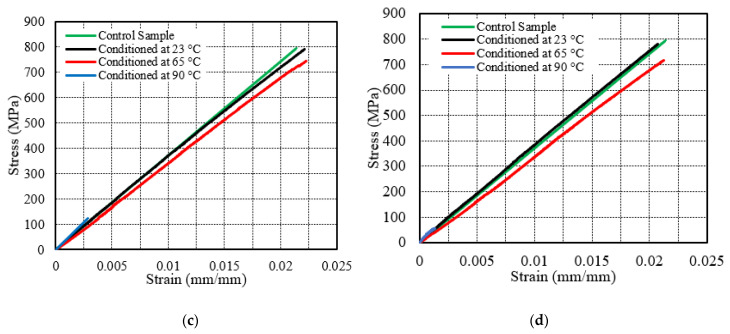
Stress–strain behavior of specimen immersed in seawater for (**a**) 3 months, (**b**) 6 months, (**c**) 9 months and (**d**) 12 months.

Further, the effect of temperature in seawater conditioning was examined by the analogy of stress–strain behavior of the sample immersed for 3 months, 6 months, 9 months and 12 months at 23 °C, 65 °C and 90 °C as shown in Figure 7. The strength of specimens conditioned in seawater at 23 °C decreased by 0.25% after 3 m, 0.5% after 6 months, 0.75% after 9 m and by 1% after 12 m from the control specimen, as shown in Figure 7a–d, respectively. Figure 7a shows that the gradient of the specimen immersed at 23 °C is more as compared to the gradient of the control specimen demonstrating the increase in tensile modulus by 1.9% from 37.1 GPa to 37.8 GPa after three months of conditioning. The slope and modulus decreased a little by 5.3% after 6 months, and 2.15% after 9 months whereas increased again by 3.8% in 12 months as indicated in Figure 7b–d, respectively. The plots also demonstrate the equivalent variation in failure strain, first declining by 1.8% then increasing by 5.7% and 3.7% and again decreasing by 0.9% after 3 months, 6 months, 9 months and 12 months respectively. At 65 °C, the strength of the specimens reduced by 1.9% after 3 months, 4.13% after 6 months, 5% after 9 months, and by 9% after 12 months as indicated in Figure 7a–d, respectively. The slopes of the graphs show the decrease in tensile modulus by 11.8% from 37.1 MPa to 32.7 MPa in a duration of 12 months. The failure strain of the specimen slightly increased by 1.8% after 12 months of conditioning at 65 °C as shown in Figure 7d. Maximum stain-at-failure observed after 6 months by 7.5% is shown in Figure 7b. At 90 °C, the tensile strength of the E-glass/epoxy composite declined by 58.9% after 3 months, 76.2% after 6 months, 86% after 9 m, and by 93.5% after 12 months as shown in Figure 7a–d, respectively. The slopes of the graphs indicate that the modulus increased by 26.7% from 37.1 MPa to 47 MPa in the duration of 12 months.

### 3.3. SEM Analysis

**Figure 8:**
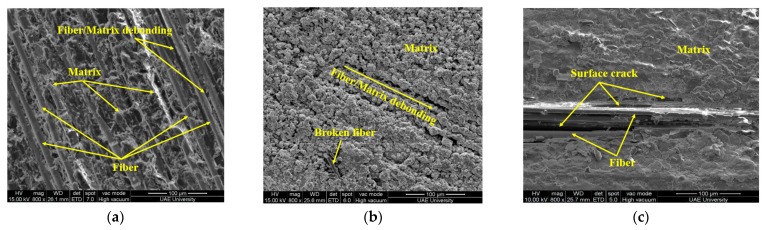
Surface micrograph of specimen immersed for the duration of one-year at (**a**) 23 °C, (**b**) 65 °C and (**c**) 90 °C.

The microstructure of samples was analyzed using JEOL-JSM 7610F (JOEL Ltd., Tokyo, Japan) scanning electron microscope. Figure 8a represents the effect of seawater at 23 °C on the surface of the e-glass epoxy composite. The bonding between fiber and matrix is slightly affected without the presence of any crack. However, minor cracks and broken fiber were detected on the surface of the sample immersed at 65 °C as shown in Figure 8b. The crack size increased with the increase of immersion temperature. At the temperature of 90 °C, shown in Figure 8c, a significantly larger crack was observed which allows the seawater to penetrate the fiber layers. This penetration may cause the strength reduced by 93% in 12 months of immersion.

**Figure 9:**
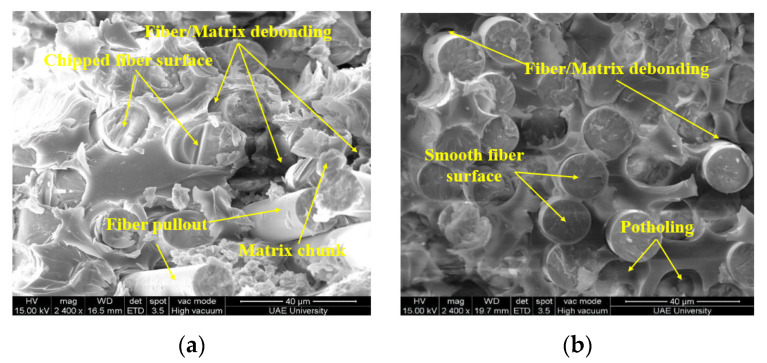
Failure surface of specimen immersed for the duration of 12 months at (**a**) 23 °C, and (**b**) 65 °C.

SEM images for the control sample compared with samples immersed at 23 °C and 65 °C mentioned in Figure 9a,b for the duration of 12 months. Rough fracture and chipping surface of fiber in Figure 9a indicates the ductile failure of the fiber. Shattered matrix and smooth fractured surface indicate brittle failure of the matrix. The microstructure of the fractured surface and fiber pullout represents slight degradation in fiber/matrix bonding. The composite indicated only a 1% decrease in tensile strength, a 3.77% increase in modulus due to swelling of the matrix and a 0.93% decrease in failure strain due to matrix plasticization. 

The micrograph in Figure 9b represents the failure surface of a specimen immersed for 12 months at 65 °C. The smooth cross-sectional surface of the fibers indicates brittle fracture of the fiber. The presence of epoxy resin around fibers and dimples on the surface of the matrix indicates ductile failure. The fiber/matrix debonding and potholing were observed at the fractured surface which results in a 9% drop in tensile strength.

**Figure 10:**
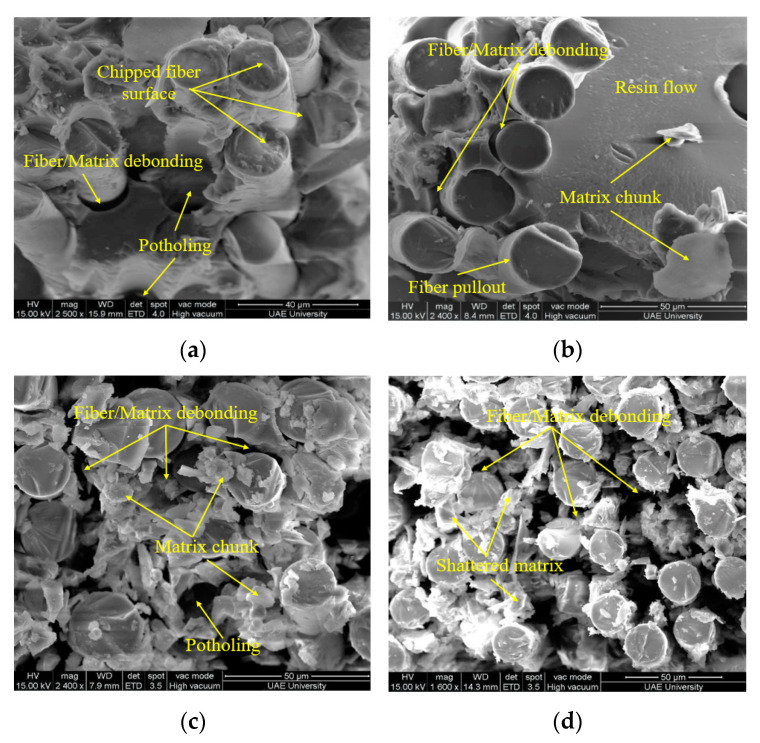
Failure surface of specimen immersed in sea water at 90 °C (**a**) 3 months, (**b**) 6 months, (**c**) 9 months and (**d**) 12 months.

Figure 10a–c show the SEM micrograph of E-glass/epoxy composite immersed at 90 °C for the duration of 3 months, 6 months, 9 months and 12 months respectively. The rough and corrugated surface of the fiber and dimpled matrix surface indicates the ductile failure of both fiber and matrix as shown in Figure 10a. The potholing and fiber/matrix debonding observed on the surface may results in a sharp decrease in the tensile strength of the composite as shown in Figure 4. With the increase of immersion time, resin flow and fiber/matrix debonding accelerated as shown in Figure 10b. The chipped-out fiber surface indicates the ductile fracture of the fiber whereas the smooth and mirror-like surface with matrix chunks indicates its brittle failure. The reaction between water and epoxy believed to cause a breakdown of the polymer’s molecular weight, leading to the fragile nature of the matrix. Water can also function as an anti-plasticizer, preventing polymer segments from moving and making the matrix more brittle [46]. After an immersion of 9 and 12 months, the fiber matrix bonding degraded to the lowest level and an absence of the resin was observed at the fractured surfaces of the composite as shown in Figure 10c,d. These SEM images support the observed decrease in the mass of the composite due to resin flow discussed in the water absorption test and shown in Figure 3. The degradation at the fiber/matrix interface involves a complex mechanism as the fiber/matrix interface is a heterogeneous area between the fiber and matrix [63,64,65,66,67]. Fiber-matrix interface damage for FRP composites is usually caused by debonding between fiber and resin which occurs mainly in two stages. The first stage is the chemical debonding due to chemical corrosion between fiber and resin, and the second stage is the poor interlacing between fibers and matrix owing to resin swelling through water absorption [68]. 

### 3.4. Differential Scanning Calorimetry (DSC) Test

**Figure 11:**
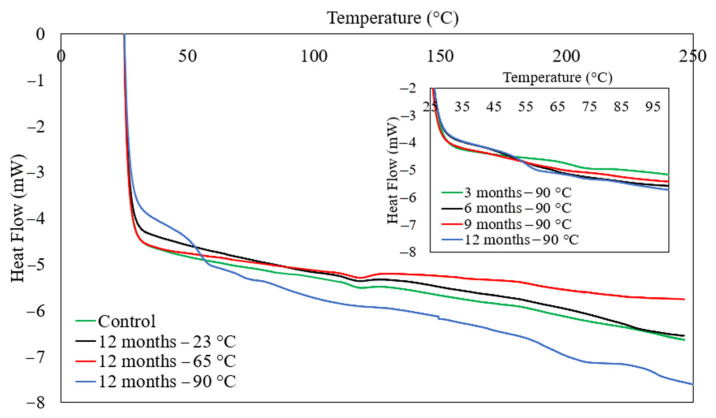
DSC curves for E-glass/epoxy control sample and conditioned samples.

The differential scanning calorimetry (DSC) for the E-glass/epoxy composite was conducted on samples immersed at different temperatures and exposure times. This test was carried out using a TA-Instruments DSC Q200 device (TA-Instruments, New Castle, DE, USA). The test was performed in an inert environment using nitrogen gas. The experiment was run between 25 °C and 250 °C with a heating rate of 10.0 °C/min. Three samples for each condition were considered to determine the glass transition temperature. Figure 11 represents the DSC curves for the control sample and specimens immersed at 23 °C, 65 °C and 90 °C for 12 months. The glass transition temperatures (Tg) of the samples immersed at 23 °C and 65 °C found to be close to the Tg of the control sample. The glass transition temperature for the control specimen and specimen immersed at 23 °C and 65 °C observed to be 118 °C, 117 °C and 112 °C, respectively, indicating that as exposure temperature increases, the Tg decreases.

This is an indication of the associated effect of immersion on the physical and chemical properties of the composite. It was noted that the tensile strength reduced by 1% and 9% in one year for the exposure temperature of 23 °C and 65 °C, respectively. Furthermore, SEM micrographs and FTIR analysis support the observed slight degradation. On the other side, the Tg of the composite was significantly reduced to 42.11 °C after seawater aging at 90 °C for 12 months. The value of Tg was observed to be 69.8 °C, 51.55 °C, 46.57 °C and 42.11 °C for the duration of 3 months, 6 months, 9 months and 12 months, respectively. This significant shift in Tg is because of the combined effect of high temperature and seawater aging. The degradation in the epoxy composite owes to the existence of hydrophilic groups which form weak hydrogen bonds by reacting with water molecules during immersion and swelling of the composite at 90 °C [69]. This demonstrates that the moisture uptake acts as a plasticizer and decreases Tg. The reduction in Tg represents thermal degradation and loss in mechanical properties which limits the service temperature of the polymer. The mechanisms of the long chain of the polymer may start to isolate at high-temperature immersion and react with each other to alter the properties of the polymer [70].

### 3.5. FTIR Results

**Figure 12:**
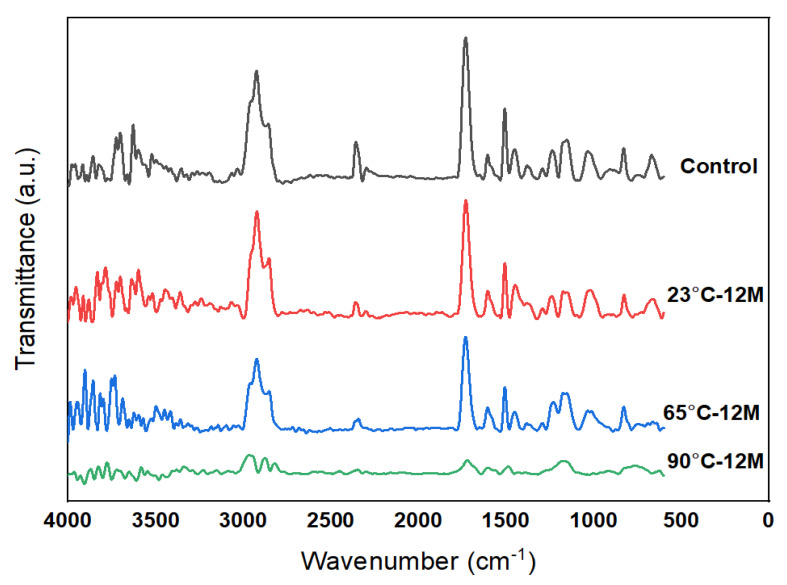
FTIR spectrum of E-glass/epoxy control sample and conditioned samples.

The Fourier transform infrared spectroscopy (FTIR) was performed on Perkin Elmer Spectrum 100 FTIR spectrometer (PerkinElmer Life and Analytical Sciences, Shelton, CT, USA) at room temperature in the transmission mode. FTIR spectra were logged in between 600 cm^−1^ and 4000 cm^−1^ at a resolution of 2 cm^−1^ with 10 scans. Before testing the samples, Background spectra were taken in the empty chamber to eliminate the influence of moisture and CO2 in air. Figure 12 presents a comparison of typical spectra for E-glass/epoxy composite immersed at 23 °C, 65 °C and 90 °C for 12 months and compared with control sample. The seawater exposure will lead to the presence of equivalent FTIR bands on the residue spectrum due to the leaching of functional groups from the resin of the composite. The allocation of the representative absorbent bands is given in Table 5 for the samples and their allocated functional groups. The O–H stretching band is observed above 3000 cm^−1^ wavenumbers.

The difference in intensity of O–H stretching vibration at 3400 cm^−1^ is due to the seawater immersion of the specimen [71,72]. The next band is located between 2930 cm^−1^ and 2900 cm^−1^ and is attributed to the stretching band of the C–H group of epoxies [71,72]. The stretching of C–O non-conjugate ester was detected at 1732 cm^−1^ [73]. The existence of bands at 1509 cm^−1^ and 1610 cm^−1^ are allotted for C=C stretching in aromatics and alkenes respectively [72,74,75]. Symmetric and asymmetric stretching vibration of C–O–Φ observed at 1040 cm^−1^ and 1245 cm^−1^ wavenumber respectively [71,73]. The band at 1182 cm^−1^ is due to the vibration characteristics of C–O in an aromatic ring [76] whereas C–H bending in benzene ring was detected at 827 cm^−1^ [72]. The immersion of specimen in seawater produces a band at ~840 cm^−1^ which indicates stretching vibration of the ether group as trapped moisture in specimen includes hydrogen bond with the C-O-C groups [72]. Another band that appeared at ~1125 cm^−1^ could be ascribed to the alteration in the vibrational C-OH band leading to the formation of hydrogen bond (C–O–H----OH2) during hydration [72]. The presence of these bands confirms the leaching of the E-glass/epoxy matrix into the seawater during immersion.

In the FTIR spectrum, two peaks at 2296.3 cm^−1^ and 2353.2 cm^−1^ appeared for the control sample. This indicates the existence of unreacted –N=C=O groups [77] in E-glass/epoxy samples. These two peaks become weak after immersion of 12 months at 23 °C and 65 °C and disappeared at 90 °C. The vanishing of the two peaks at 2296.3 cm^−1^ and 2353.2 cm^−1^ after immersion for 12 months at 90 °C is due to the reaction between the water molecules and unreacted –N=C=O groups, which can be presented shown as

(2) $$R-N=C=O+H_{2}O\to R-{\mathrm{NH}}_{2}+{\mathrm{CO}}_{2}↑$$

Therefore, the mass loss of the E-glass epoxy composite specimens during immersion at 90 °C can be described as the release of CO_2_ shown in Equation (2). At this immersion temperature, the sustained release of CO_2_ is responsible for the increase of mass loss of the composite [67,78,79].

## 4. Prediction

Several researchers utilized the Arrhenius method for the prediction of the long-term behavior of FRP composites [48,80,81]. A fundamental hypothesis was anticipated that the single dominant degradation mechanism does not alter irrespective of temperature and exposure time [33], but the damage resistance reduces with the exposure temperatures.

The equation for the degradation rate is mentioned below [82];

(3) k=A e(−EaRT)

where *k* is the rate of degradation (1/time), *A* is the constant of material in the degradation process, *E_a_* is the activation energy (kJ/mol), *R* is the universal gas constant (8.314 Jmol^−1^K^−1^), *T* is the temperature (K). Equation (3) can be modified as:

(4) $$\frac{1}{k}=\frac{1}{A}\;e^{\left(\frac{E_{a}}{RT\;}\right)}$$

(5) $$\ln\left(\frac{1}{k}\right)=\frac{E_{a}}{R}\frac{1}{T}-\ln\left(A\right)$$

**Figure 13:**
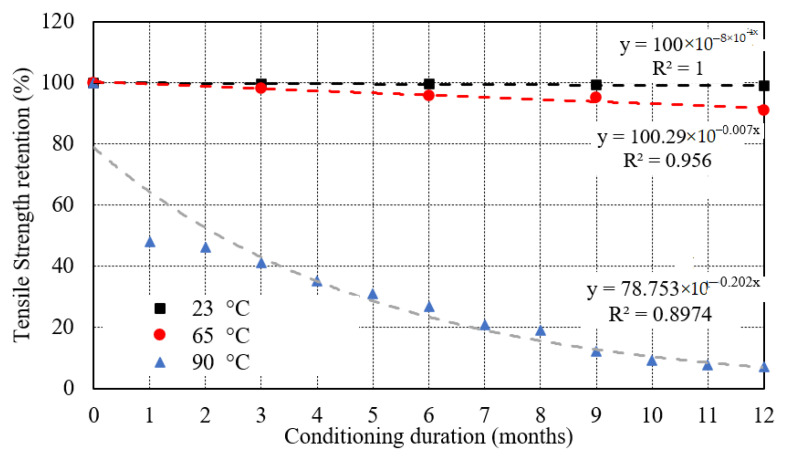
Tensile strength retention of E-glass epoxy samples exposed to seawater at 23 °C, 65 °C, and 90 °C.

The inverse of *k* indicates the time taken to achieve a given value for a material property. The exponential degradation model [83] was utilized as the fiber-matrix interfacial delamination took place during the seawater immersion of E-glass/epoxy composite (Figure 13). This model can be expressed as

(6) $$Y=100e^{\left(\frac{-t}{\tau }\right)}$$

where *Y* is the tensile strength retention (%), *τ* is a constant and *t* is the immersion time (month). the value of correlation coefficient (R^2^) and *τ* of E-glass/epoxy composite are mentioned in Table 6 by fitting Figure 13 using Equation (6). 

A different approach based on a time-shift factor (TSF) was used by Dejke [49]. The time-shift is defined as the ratio of times (t_1_ and t_2_) required to determine a certain level of decrease in the mechanical property at two different temperatures (T_1_ and T_2_). According to Equation (7), the time required to obtain a certain level of decrease in mechanical property is the inverse proportion of the reaction rate K.

(7) TSF=t1t2=(1−Y)K1(1−Y)K2=K2K1=Ae−EaT2RAe−EaT1R=eEaR(1T2−1T1)

This approach also depends on the Arrhenius equation. The time shift is an effect of accelerated aging due to the increment in temperature from T_2_ (lower temperature) to T_1_ (higher temperature) [84]. The TSF was calculated, using Equation (7), shown in Table 7 for 65 °C and 90 °C. 

**Figure 14:**
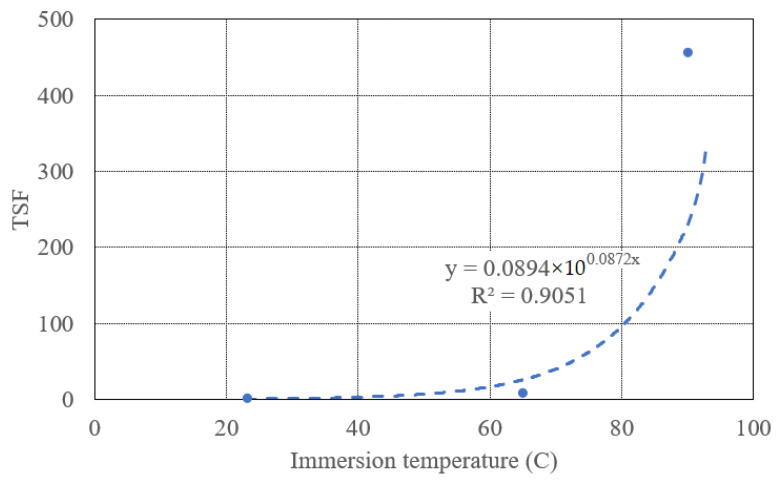
The TSF values versus temperature for E-glass epoxy composite.

The value of Ea/R can also be calculated using the time-shift approach by plotting the TSF versus the temperature (in °C) graph as shown in Figure 14 and fitted by regression analysis so that the fit is given by Equation (7). The values of Ea/R are presented in Table 7.

**Figure 15:**
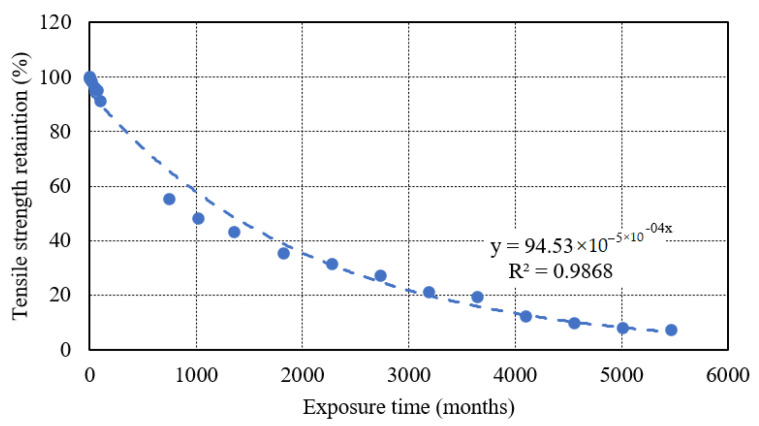
Durability prediction of E-glass epoxy composite immersed in seawater at 23 °C.

The durability of composite with a reference temperature of 23 °C calculated by time-shift from 23 °C to 65 °C and 23 °C to 90 °C. The TSF values calculated from Equation (7) using temperatures 23 °C and 90 °C is 456 and the time t_2_ to reach 57% of the strength at 90 °C is 12 months, then the time t_1_ to reach 7.2% strength at 23 °C is 456 × 12 = 5472 months. The extrapolations to predict the long-term tensile strength retention at 23 °C from the strength retention at 65 °C and 90 °C based on the time-shift approach for E-glass/epoxy are shown in Figure 15. The discussed method can be utilized to predict the durability of any structure under seawater at any temperature. In contrast, E-glass/epoxy possesses a long-term performance in tensile strength under seawater immersion at 23 °C. However, at an accelerated temperature of 90 °C, the tensile and chemical properties of the composite degraded significantly in a short period of immersion which restricts the application of the composite at high-temperature immersion.

## 5. Conclusions

In this study, E-glass/epoxy composites were conditioned at different exposure temperatures (23 °C, 65 °C and 90 °C) in Gulf seawater for a period of 12 months. The water absorption, tensile properties and failure analysis were conducted and the durability of the composite predicted using the TSF approach. The outcomes of the investigation are summarized below:

(1) The mass of the specimen increased by 2.5% and 5% after the immersion of 12 months at 23 °C and 65 °C respectively. An opposite tendency was observed for the specimen immersed at 90 °C in which the mass of the sample reduced by 12.7% for the same immersion period.
(2) The tensile strength reduced by 1% and 9% after immersion of 12 months at 23 °C and 65 °C respectively. The durability of the composite was significantly affected at at 90 °C and tensile strength was reduced to 48.4% in 1 month.
(3) Slight variation in the tensile modulus observed for specimen immersed at 23 °C and 65 °C where it increased significantly at 90 °C. However, the failure strain slightly increased for specimens at 23 °C and 65 °C but it decreased drastically for the immersion at 90 °C.
(4) SEM micrographs indicate fiber/matrix debonding, potholing, fiber pull-out and matrix cracking which indicates deterioration in the tensile properties of the composite. The deterioration mainly owes to breakage of chemical bonding between fiber and resin due to chemical corrosion, and poor interlocking between fibers and resin due to resin swelling through water absorption especially at 90 °C immersion, mainly from the hydrolysis of resin, which was also evidenced by the DSC and FTIR results.
(5) A prediction approach based on a time-shift factor (TSF) was used which utilizes the accelerated temperature testing results to build a model for the long-term prediction at room temperature. This model predicted that the tensile strength retention of E-glass/Epoxy composite will be reduced to 7% 450 years after immersion in seawater at 23 °C. Lastly, the activation energy for the degradation of the composite was calculated. It was 5155.713 and 9731.482 for composite immersed at 65 °C and 90 °C respectively.

It can also be concluded that the E-glass/epoxy composite showed outstanding performance in seawater immersion up to 65 °C. However, the rate of degradation was significantly high for immersion at 90 °C and the material almost lost its durability. This is an indicative measure that the durability of the E-glass/epoxy composite is uncertain above 65 °C which may limit its utilization for high-temperature applications.

## Figures and Tables

**Table 1:** FRP composite immersed at different environmental conditions.

Authors	Composite	Conditioning	Duration
Silva et al. [45]	E-glass/epoxy	Saltwater at 30 °C, 45 °C and 55 °C	750–5000 h (Approx 7 months)
Chakraverty et al. [46]	E-glass/epoxy	Seawater at room temperature	2, 4, 6, 8, 10, and12 months (1 year)
Hu et al. [50]	Glass/polydicycl-opentadiene and glass/epoxy	Saltwater and deionized water at 60 °C	1, 3, 6, and 12 months (1 year)
Guermazi et al. [51]	Glass/epoxy, carbon/epoxy and glass/carbon/epoxy	Tap water at 24 ± 3, 70 and 90 °C	3 months
Bobba et al. [52]	E-glass and S-glass fiber-epoxy	Tap water at 90 °C	600, 1200, and 1800 h (2.5 months)
Feng et al. [53]	Glass/epoxy	H_2_SO_4_, NaOH and NaCl at 60 and 90 °C	7, 15, 30, and 90 days (3 months)
Merah et al. [54]	Glass fiber-reinforced epoxy (GFRE)	Seawater at outdoor temperature	6 and 12 months
Mourad et al. [18]	Glass/epoxy and glass/polyurethane	Seawater at 23 °C and 65 °C	3, 6, 9, and 12 months
EminDeniz et al. [55]	Glass/epoxy composite	Seawater at 20 °C	3, 6, 9, and 12 months
Pavan et al. [43]	E-glass/epoxy laminates	Artificial seawater in sub-zero and ambient temperatures	3600 h (5 months)
Wei et al. [56]	Basalt fiber-reinforced plastic (BFRP) and Glass fiber-reinforced plastic (GFRP)	Artificial seawater at 25 °C	10, 20, 30, 60, and 90 days
Antunes et al. [57]	Glass/epoxy filament wound cylinders	Seawater at 80 °C	7–28 days
Ghabezi et al. [58]	Carbon/epoxy and glass/epoxy	Artificial seawater at room temperature and 60 °C	45 days

**Table 2:** Percentage change in mass of E-glass/epoxy specimens.

Conditioning Duration (Months)	Variation in Mass (%)		
	23 °C	65 °C	90 °C
3	0.9 ± 0.2	2.8 ± 0.3	1.07 ± 0.14
6	1.3 ± 0.4	2.9 ± 0.2	3.6 ± 0.9
9	1.7 ± 0.7	4.1 ± 0.6	5.9 ± 0.65
12	2.5 ± 0.5	5.0 ± 0.8	12.05 ± 1.67

**Table 3:** Tensile properties of E-glass/epoxy composite immersed in seawater at 23 °C and 65 °C.

Immersion Time (Months)	Tensile Strength (MPa)		Tensile Modulus (GPa)		Tensile Strain to Failure (%)	
	23 °C	65 °C	23 °C	65 °C	23 °C	65 °C
0 (Control Sample)	798 ± 43	798 ± 43	37.1 ± 2.5	37.1 ± 2.5	2.14 ± 0.02	2.14 ± 0.02
3	796 ± 40	783 ± 51	37.8 ± 1.6	35.4 ± 1.8	2.1 ± 0.2	2.2 ± 0.0
6	794 ± 49	765 ± 17	35.1 ± 2.5	33.5 ± 0.6	2.26 ± 0.03	2.3 ± 0.2
9	793 ± 39	758 ± 28	36.3 ± 1.9	33.7 ± 2.3	2.22 ± 0.2	2.24 ± 0.15
12	790 ± 38	726 ± 39	38.5 ± 4.5	32.7 ± 3.4	2.12 ± 0.21	2.18 ± 0.1

**Table 4:** Tensile properties of the specimen immersed in seawater at 90 °C.

Immersion Time (Months)	Tensile Strength (MPa)	Tensile Strain to Failure (%)	Tensile Modulus (GPa)
0 (Control sample)	798 ± 43	2.14 ± 0.02	37.1 ± 2.5
1	384 ± 55	1.27 ± 0.02	35.49 ± 2.13
2	368.7 ± 51	1.16 ± 0.023	34.07 ± 1.27
3	328.2 ± 19	0.88 ± 0.08	41.87 ± 2.48
4	279.9 ± 9	0.87 ± 0.07	42.79 ± 4.05
5	248 ± 27	0.75 ± 0.1	41.50 ± 2.81
6	215 ± 16	0.51 ± 0.14	40.43 ± 1.23
7	165.6 ± 14	0.31 ± 0.09	43.35 ± 4.33
8	151.9 ± 17	0.40 ± 0.6	42.1 ± 3.9
9	96.3 ± 26	0.3 ± 0.1	43.8 ± 2.8
10	74.7 ± 9	0.27 ± 0.11	47.38 ± 2.8
11	60.5 ± 10	0.15 ± 0.07	47.18 ± 1.13
12	57.3 ± 3.7	0.14 ± 0.09	46.9 ± 4.7

**Table 5:** FTIR bands observed in E-glass/epoxy specimens.

Bands (cm^−1^)	Assignment
3400	Stretching vibration O=H
~2930 and ~2900	C–H group stretching band
~1732	C–O non-conjugate ester stretching
~1610	stretching band of C=C (alkene)
~1509	C=C (aromatic nucleus)
~1245	Asymmetric C–O– Φ stretch
1182	C–O aromatic ring stretching
~1040	Symmetric C–O– Φ stretch
~827	Out of plane bending of C–H (benzene)

**Table 6:** Coefficients of regression equation.

Temperature	τ	R^2^
23	1250	1
65	142.86	0.9544
90	4.95	0.966

**Table 7:** Value of time-shift factor and activation energy.

Temperature	TSF	E_a_/R
65	8.7	5155.713
90	456	9731.482

## Data Availability

All data and models used during the study appear in the submitted article.
